# Evaluation of the Direct Costs of Managing Adverse Drug Events in all Ages and of Avoidable Adverse Drug Events in Older Adults in Japan

**DOI:** 10.3389/fphar.2021.761607

**Published:** 2021-11-17

**Authors:** Hayato Katsuno, Tomoya Tachi, Takuya Matsuyama, Mayuko Sugioka, Satoshi Aoyama, Tomohiro Osawa, Yoshihiro Noguchi, Masahiro Yasuda, Chitoshi Goto, Takashi Mizui, Hitomi Teramachi

**Affiliations:** ^1^ Laboratory of Clinical Pharmacy, Gifu Pharmaceutical University, Gifu, Japan; ^2^ Department of Pharmacy, Gifu Municipal Hospital, Gifu, Japan; ^3^ Laboratory of Community Health Pharmacy, Gifu Pharmaceutical University, Gifu, Japan

**Keywords:** adverse drug event, direct cost, inpatient, older adult, outpatient

## Abstract

In Japan, medical costs are increasing annually, and the increase in national medical costs, particularly in the direct cost of managing adverse drug events, is high. An in-depth understanding of these costs is important for their reduction. This study aimed to calculate the direct cost of managing adverse drug events in all ages, including older adults, and that of avoidable adverse drug events in older adults. We conducted a retrospective survey on patients aged 1 year or older who visited Gifu Municipal Hospital in Japan. We investigated and calculated the direct cost of managing adverse drug events and that of avoidable adverse drug events based on the Beers Criteria Japanese version (BCJ) and “Guidelines for medical treatment and its safety in the elderly 2015” (GMTSE-2015) in inpatients and outpatients. Among 6,504 patients, 11.1% visited the hospital or were hospitalized due to adverse drug events. The direct costs per patient with adverse drug events were 21,281 and 22,590 yen (166 and 176 euros as on September 13, 2021) for outpatients, and 853,175 and 874,582 yen (6,648 and 6,815 euros) for inpatients of all ages and older adults, respectively. The direct costs of avoidable adverse drug events per patient using drugs listed in the BCJ and GMTSE-2015 for older adults were 3,212 and 3,341 yen (25 and 26 euros) for outpatients, and 55,548 and 80,246 yen (433 and 625 euros) for inpatients, respectively. In sum, considering both inpatients and outpatients in the whole country, the direct costs of managing adverse drug events were 804.53 billion and 597.19 billion yen (6,269 million and 4,653 million euros) per year for all ages and older ages, respectively. The direct cost of avoidable adverse drug events in older adults was 83.43–258.44 billion yen (650–2,013 million euros) per year. We found that, in Japan, high medical costs are often caused by managing adverse drug events, and that the costs of avoidable adverse drug events in older adults based on the BCJ and GMTSE-2015 account for a substantial proportion of the medical cost. Therefore, by using the BCJ and GMTSE-2015, avoiding adverse drug events and reducing medical costs may be possible.

## Introduction

Globally, medical costs are increasing annually (The Organisation for Economic Co-operation and Development, 2021). In the United States and Japan, the national medical costs in 2015 were 3,051,508 million dollars and 42,364.4 billion yen, representing an increase of 5.9 and 3.8% from the previous year, respectively (Ministry of Health, Labour and Welfare in Japan, 2015; The Organisation for Economic Co-operation and Development, 2021). In Japan, medical costs for adults aged 65 and over, which were 25,127.6 billion yen, increased by 5.1% from the previous year, and those for older adults exceeded 50% of the total medical costs (Ministry of Health, Labour and Welfare in Japan, 2015). Thus, measures to reduce these ever-increasing medical costs should be taken.

A substantial proportion of hospital consultations is attributable to adverse drug events. During 1990–2020, the proportion of outpatient visits and hospitalizations due to adverse drug events has varied from 5.5 to 35.0% (Hanlon et al., 1997; Honigman et al., 2001; Gandhi et al., 2003) and 1.3 to 30.4% (Dartnell et al., 1996; Chan et al., 2001; Senst et al., 2001; Onder et al., 2002; Wawruch et al., 2009; Ruiter et al., 2012; Parameswaran Nair et al., 2017), respectively. In Japan, 1.7% of hospitalizations are attributable to inappropriate drug use (Koinuma et al., 2006). The prevalence of adverse drug events resulting in hospital consultations is a relevant factor of the high costs of managing adverse drug events in hospitals. Therefore, every country calculates the direct costs of managing adverse drug events, including treatment and examination, which were evaluated to be quite high (Bates et al., 1997; Carrasco-Garrido et al., 2010; Leendertse et al., 2011; Rottenkolber et al., 2011; Stark et al., 2011). However, there are no data for Japan; therefore, it is important to evaluate the cost in Japan. It is important to clarify the costs of managing adverse drug events and to evaluate the contribution of the costs to overall medical costs.

Studies have been conducted to determine which drugs should be avoided and discontinued by older adults (Opondo et al., 2012; Wickop and Langebrake, 2014; Lucchetti and Lucchetti, 2017; Nothelle et al., 2017), and results have shown that 2.9–38.5% of older adults’ prescriptions are potentially inappropriate (Opondo et al., 2012). To determine which drugs should be avoided and discontinued by the elderly, the Beers Criteria Japanese version (BCJ) was published by the National Institute of Public Health in 2008 (National Institute of Public Health, 2010), and the “Guidelines for medical treatment and its safety in the elderly 2015” (hereinafter referred to as GMTSE-2015) was published by the Japan Geriatrics Society in 2015 (The Japan Geriatrics Society, 2015; Kojima et al., 2016). In the previous study, we investigated the prevalence of older patients targeted by the BCJ and GMTSE-2015, and we clarified the prevalence and background/factor of adverse drug events occurred (Tachi et al., 2019). The avoidance of adverse drug events by appropriate prescriptions results in a reduction of cost of managing adverse drug events. The calculation of costs of preventable adverse drug events is important to understand its effect on overall medical costs. This calculated cost has been reported (Rothschild et al., 2002; Leendertse et al., 2011; Slight et al., 2018). The costs that can be reduced by using the criteria for drugs that should be avoided and considered for discontinuation by older adults, such as those listed in the BCJ and GMTSE-2015, should also be identified in Japan.

We conducted a retrospective survey with patients who visited a hospital in Japan to calculate the direct cost of managing adverse drug events. Further, we calculated the degree of this reduction using the BCJ and GMTSE-2015 as references.

## Materials and Methods

### Participants

The target patients were outpatients and inpatients aged at least 1 year who visited Gifu Municipal Hospital (Gifu, Japan), excluding outpatients and inpatients with reservations, between July 1 and December 31, 2015, and took one or more drugs, excluding investigational drugs, during the visit. Patients hospitalized immediately after their hospital visit as outpatients were included as inpatients.

With 609 beds, Gifu Municipal Hospital is a typical general hospital in Japan, providing primary and secondary care to the city of Gifu and its suburbs.

### Investigations and Evaluations

The survey was conducted retrospectively using electronic medical records. The survey items were sex, age, drugs used at the time of hospital visit or hospitalization, disease, presence or absence of adverse drug events, and length of hospitalization in case of inpatients. For people aged 65 years and above, we investigated whether they used the drugs listed in the BCJ and GMTSE-2015.

Drugs were classified according to the YJ code (unique code for each item listed on the NHI drug price standard) used in Japanese insurance claims. Meanwhile, diseases were classified using the International Statistical Classification of Diseases and Related Health Problems 10th Revision (ICD-10) (World Health Organization, 2016).

Adverse drug events were extracted in accordance with the Global Trigger Tool (Institute for Healthcare Improvement, 2003), and events’ severity was evaluated using the Common Terminology Criteria for Adverse Events Version 5.0 (Japan Clinical Oncology Group, 2017). The causality was categorized into “possible,” “probable/likely,” and “certain,” using the Causality Assessment System published by Uppsala Monitoring Centre (UMC) (World Health Organization – Uppsala Monitoring Centre, 2012). Two pharmacists with at least 10 years of clinical experience and, where necessary, a physician determined these adverse drug events and classified their severity and causality.

### Calculation of Direct Costs of Managing Adverse Drug Events

In this study, we evaluated direct costs from the perspective of medical cost payers under the public medical insurance system. The direct costs of managing adverse drug events were evaluated in outpatients who visited the hospital due to such events and inpatients who were admitted to the hospital due to the same. Direct costs due to avoidable adverse drug events in older adults were evaluated in older patients who visited (outpatients) or were admitted to (inpatients) the hospital due to adverse drug events caused by the drugs listed in the BCJ and GMTSE-2015. For those who visited or were admitted to the hospital for reasons other than adverse drug events, the direct costs were recorded as 0 yen.

We calculated the direct costs by stratifying outpatients and inpatients because information on the numbers of outpatients and inpatients is available in Japan’s domestic statistical data (Ministry of Health, Labour and Welfare in Japan, 2014). The direct costs were extrapolated to the national level (whole country) by linear regression, based on an overview of the 2014 patient survey (Ministry of Health, Labour and Welfare in Japan, 2014). Please see the detailed calculations in Supplementary Material.

#### Direct Costs of Managing Adverse Drug Events in Outpatients

For all ages, older adults (>65-year-olds), and <65-year-olds, we calculated the direct costs of managing adverse drug events per patient who visited the hospital due to these events (the sum of the direct cost for outpatients with adverse drug events divided by the number of outpatients with adverse drug events) (*C*_*out, ade*_). In addition, we calculated the direct costs of managing adverse drug events per patient who visited the hospital regardless of the visit reason (the sum of the direct cost for outpatients with adverse drug events divided by the number of all outpatients targeted in this study) (*C*_*out, targ*_). We then obtained the direct costs of managing adverse drug events in outpatients in the whole country (*C*_*out, nat*_) using Equation 1; (Table 1).

**TABLE 1:** Calculation of costs.

Equation 1	$Cout,\;nat=Cout,\;targ\times \left[\frac{\left(Nout,\;nat-Nout,\;nat,\;0y\right)\times 365\times Nout,\;targ}{Nout,\;hosp}\right]$
Equation 2	$Cin,\;nat=Cin,\;targ\times \left[\frac{\left(Nin,\;nat-Nin,\;nat,\;0y\right)\times 365\times Nin,\;targ}{31.9\times Nin,\;hosp}\right]$
Equation 3	$Cout,\;nat,\;BCJ=Cout,\;targ,\;BCJ\times \left(\frac{Nout,\;nat,\;older\times 365\times Nout,\;targ,\;BCJ}{Nout,\;hosp,\;older}\right)$
Equation 4	$Cout,\;nat,\;GMTSE=Cout,\;targ,\;GMTSE\times \left(\frac{Nout,\;nat,\;older\times 365\times Nout,\;targ,\;GMTSE}{Nout,\;hosp,\;older}\right)$
Equation 5	$Cin,\;nat,\;BCJ=\;Cin,\;targ,\;BCJ\times \left(\frac{Nin,\;nat,\;older\times 365\times Nin,\;targ,\;BCJ}{41.7\times Nin,\;hosp,\;older}\right)$
Equation 6	$Cin,\;nat,\;GMTSE=Cin,\;targ,\;GMTSE\times \left(\frac{Nin,\;nat,\;older\times 365\times Nin,\;targ,\;GMTSE}{41.7\times Nin,\;hosp,\;older}\right)$

[Equation 1]: *C*_*out, nat*_, the direct costs of managing adverse drug events in outpatients in the whole country; *C*_*out, targ*_, the direct costs of managing adverse drug events per patient who visited the hospital regardless of the visit reasons; *N*_*out, nat*_, the total number of outpatients on the overview of the 2014 patient survey; *N*_*out, nat, 0y*_, the total number of 0-year-old outpatients on the overview of the 2014 patient survey; *N*_*out, targ*_, the number of outpatients with more than one drug used during their hospital visit without reservation between July 1 and December 31, 2015; *N*_*out, hosp*_, the number of outpatients aged 1 year or older who visited the hospital between July 1 and December 31, 2015.

[Equation 2]: *C*_*in, nat*_, the direct costs of managing adverse drug events in inpatients in the whole country; *C*_*in, targ*_, the direct costs of managing adverse drug events per patient admitted to the hospital regardless of the reasons; *N*_*in, nat*_, the total number of inpatients on the overview of the 2014 patient survey; *N*_*in, nat, 0y*_, the total number of 0-year-old inpatients on the overview of the 2014 patient survey; *N*_*in, targ*_, the number of inpatients with more than one drug used at their hospitalization without reservation between July 1 and December 31, 2015; *N*_*in, hosp*_, the number of inpatients aged 1 year or older who were admitted to the hospital between July 1 and December 31, 2015.

[Equations 3 and 4]: *C*_*out, nat, BCJ*_ and *C*_*out, nat,*_ _*GMTSE*_; the direct costs of avoidable adverse drug events based on the BCJ and GMTSE in older outpatients in the whole country, respectively; *C*_*out, targ, BCJ*_ and *C*_*out, targ, GMTSE*_, the direct costs of avoidable adverse drug events per older outpatient using the drugs listed in the BCJ and GMTSE regardless of the visit reason, respectively; *N*_*out, nat, older*_, the number of over-65-year-old outpatients in a day on the overview of the 2014 patient survey; *N*_*out, targ, BCJ*_ and *N*_*out, targ, GMTSE*_, the number of older outpatients using more than one drugs listed in the BCJ and GMTSE during their hospital visit without reservation between July 1 and December 31, 2015, respectively; *N*_*out, hosp, older*_, the number of older outpatients who visited the hospital between July 1 and December 31, 2015.

[Equations 5 and 6]: *C*_*in, nat, BCJ*_ and *C*_*in, nat, GMTSE*_, the direct costs of avoidable adverse drug events based on the BCJ and GMTSE-2015 in older inpatients in the whole country, respectively; *C*_*in, targ, BCJ*_ and *C*_*in, targ, GMTSE*_, the direct costs of avoidable adverse drug events per older inpatient using the drugs listed in the BCJ and GMTSE-2015 regardless of the hospitalization reason, respectively; *N*_*in, nat, older*_, the number of over-65-year-old inpatients on the overview of the 2014 patient survey; *N*_*in, targ, BCJ*_ and *N*_*in, targ, GMTSE*_, the number of older inpatients using more than one drug listed in the BCJ and GMTSE-2015 at their hospitalization without reservation between July 1 and December 31, 2015, respectively; *N*_*in, hosp, older*_, the number of older patients who were admitted to the hospital between July 1 and December 31, 2015.

#### Direct Costs of Managing Adverse Drug Events in Inpatients

For all ages, older adults, and <65-year-olds, we calculated the direct costs of managing adverse drug events per patient admitted to the hospital due to these events (the sum of the direct cost for inpatients with adverse drug events divided by the number of outpatients with adverse drug events) (*C*_*in, ade*_). In addition, we calculated the direct costs of managing adverse drug events per patient admitted to the hospital regardless of the reason (the sum of the direct cost for inpatients with adverse drug events divided by number of all inpatients targeted in this study) (*C*_*in, targ*_). When then obtained the direct costs of managing adverse drug events in inpatients in the whole country (*C*_*in, nat*_) using Equation 2; (Table 1).

#### Direct Cost of Avoidable Adverse Drug Events in Older Outpatients

For the older outpatients who used the drugs listed in the BCJ and GMTSE-2015, we calculated the direct costs of avoidable adverse drug events per older outpatient using the drugs listed in the BCJ and GMTSE-2015 regardless of the visit reason (the sum of the direct cost in older outpatients with adverse drug events due to the drugs listed in the BCJ and GMTSE-2015 divided by the number of all the older outpatients who used the drugs listed in the BCJ and GMTSE-2015 targeted in this study) (*C*_*out, targ, BCJ*_ and *C*_*out, targ, GMTSE*_, respectively). We then obtained the direct costs of avoidable adverse drug events based on the BCJ and GMTSE-2015 in older outpatients in the whole country [*C*_*out, nat, BCJ*_ and *C*_*out, nat, GMTSE*_) using Equations 3 and 4] (Table 1).

#### Direct Cost of Avoidable Adverse Drug Events in Older Inpatients

For the older inpatients who used the drugs listed in the BCJ and GMTSE-2015, we calculated the direct costs of avoidable adverse drug events per older inpatient using the drugs listed in the BCJ and GMTSE-2015 regardless of the hospitalization reason (the sum of the direct cost of adverse drug events in older inpatients due to the drugs listed in the BCJ and GMTSE-2015 divided by the number of all the older inpatients targeted in this study who used the drugs listed in the BCJ and GMTSE-2015) (*C*_*in, targ, BCJ*_ and *C*_*in, targ, GMTSE*_, respectively). We then obtained the direct costs of avoidable adverse drug events based on the BCJ and GMTSE-2015 in older inpatients in the whole country (*C*_*in, nat, BCJ*_ and *C*_*in, nat, GMTSE*_) using Equations 5 and 6; (Table 1).

### Ethical Considerations

This study was approved by the ethics committee of Gifu Municipal Hospital (approval number: 349) and Gifu Pharmaceutical University (approval number: 28–8).

## Results

### Analysis for All Ages, Older Adults, and <65-Year-Olds

#### Patient Characteristics

Table 2 shows the patient characteristics for all ages, older adults, and <65-year-olds. Among all ages, 49.4% were male, the age was 51.2 ± 29.9 years (mean ± standard deviation), and the number of drugs used was 5.3 ± 4.2. The most common disease was “diseases of the digestive system” (53.6%). Among older adults, 50.1% were male, the age was 78.0 ± 7.5 years, and the number of drugs used was 6.8 ± 4.2. The most common disease was “diseases of the circulatory system” (76.5%).

**TABLE 2:** Patient characteristics.

	All			Older adults			<65-year-olds		
	All (n = 6,504)	Adverse drug events at the time of hospital visits and hospitalization		All (n = 3,011)	Adverse drug events at the time of hospital visits and hospitalization		All (n = 3,493)	Adverse drug events at the time of hospital visits and hospitalization	
		Present	Absent		Present	Absent		Present	Absent
		(n = 720)	(n = 5,784)		(n = 463)	(n = 2,548)		(n = 257)	(n = 3,236)
Sex [n (%)]									
Male	3,210 (49.4)	348 (48.3)	2,862 (49.5)	1,509 (50.1)	242 (52.3)	1,267 (49.7)	1,701 (48.7)	106 (41.2)	1,595 (49.3)
Female	3,294 (50.6)	372 (51.7)	2,922 (50.5)	1,502 (49.9)	221 (47.7)	1,281 (50.3)	1,792 (51.3)	151 (58.8)	1,641 (50.7)
Age (average ± standard deviation)	51.2 ± 29.9	64.4 ± 22.8	49.6 ± 30.3	78.0 ± 7.5	78.3 ± 7.6	77.9 ± 7.5	28.1 ± 21.6	39.4 ± 19.6	27.2 ± 21.5
Number of drugs used (average ± standard deviation)	5.3 ± 4.2	8.0 ± 4.7	5.0 ± 4.1	6.8 ± 4.2	8.5 ± 4.3	6.5 ± 4.1	4.0 ± 3.8	7.0 ± 5.1	3.7 ± 3.5
Inpatients [n (%)]	2,501 (38.5)	359 (49.9)	2,142 (37.0)	1,536 (51.0)	272 (58.7)	1,264 (49.6)	965 (27.6)	87 (33.9)	878 (27.1)
Length of hospitalization [days, median (range)]	11 (1–1,048)	13 (1–130)	11 (1–1,048)	15 (1–1,048)	14 (1–130)	15 (1–1,048)	7 (1–535)	9 (2–119)	6 (1–535)
Disease [n (%)]									
Certain infectious and parasitic diseases	1,517 (23.3)	185 (25.7)	1,332 (23.0)	673 (22.4)	100 (21.6)	573 (22.5)	844 (24.2)	85 (33.1)	759 (23.5)
Neoplasms	1,058 (16.3)	211 (29.3)	847 (14.6)	797 (26.5)	156 (33.7)	641 (25.2)	261 (7.5)	55 (21.4)	206 (6.4)
Diseases of the blood and blood-forming organs and certain disorders involving the immune mechanism	1,147 (17.6)	198 (27.5)	949 (16.4)	680 (22.6)	136 (29.4)	544 (21.4)	467 (13.4)	62 (24.1)	405 (12.5)
Endocrine, nutritional and metabolic diseases	2,739 (42.1)	415(57.6)	2,324 (40.2)	1,742 (57.9)	294 (63.5)	1,448 (56.8)	997 (28.5)	121 (47.1)	876 (27.1)
Mental and behavioral disorders	1,244 (19.1)	176 (24.4)	1,068 (18.5)	572 (19.0)	91 (19.7)	481 (18.9)	672 (19.2)	85 (33.1)	587 (18.1)
Diseases of the nervous system	1,661 (25.5)	233 (32.4)	1,428 (24.7)	915 (30.4)	137 (29.6)	778 (30.5)	746 (21.4)	96 (37.4)	650 (20.1)
Diseases of the eye and adnexa	1,228 (18.9)	190 (26.4)	1,038 (17.9)	802 (26.6)	136 (29.4)	666 (26.1)	426 (12.2)	54 (21.0)	372 (11.5)
Diseases of the ear and mastoid process	540 (8.3)	63 (8.8)	477 (8.2)	280 (9.3)	34 (7.3)	246 (9.7)	260 (7.4)	29 (11.3)	231 (7.1)
Diseases of the circulatory system	3,074 (47.3)	478 (66.4)	2,596 (44.9)	2,302 (76.5)	370 (79.9)	1,932 (75.8)	772 (22.1)	108 (42.0)	664 (20.5)
Diseases of the respiratory system	3,393 (52.2)	326 (45.3)	3,067 (53.0)	1,367 (45.4)	189 (40.8)	1,178 (46.2)	2,026 (58.0)	137 (53.3)	1,889 (58.4)
Diseases of the digestive system	3,486 (53.6)	487 (67.6)	2,999 (51.8)	2,027 (67.3)	328 (70.8)	1,699 (66.7)	1,459 (41.8)	159 (61.9)	1,300 (40.2)
Diseases of the skin and subcutaneous tissue	1,555 (23.9)	185 (25.7)	1,370 (23.7)	767 (25.5)	104 (22.5)	663 (26.0)	788 (22.6)	81 (31.5)	707 (21.8)
Diseases of the musculoskeletal system and connective tissue	1,742 (26.8)	244 (33.9)	1,498 (25.9)	1,076 (35.7)	168 (36.3)	908 (35.6)	666 (19.1)	76 (29.6)	590 (18.2)
Diseases of the genitourinary system	1,916 (29.5)	279 (38.8)	1,637 (28.3)	1,223 (40.6)	192 (41.5)	1,031 (40.5)	693 (19.8)	87 (33.9)	606 (18.7)
Pregnancy, childbirth and the puerperium	99 (1.5)	3 (0.4)	96 (1.7)	0 (0.0)	0 (0.0)	0 (0.0)	99 (2.8)	3 (1.2)	96 (3.0)
Certain conditions originating in the perinatal period	29 (0.4)	1 (0.1)	28 (0.5)	0 (0.0)	0 (0.0)	0 (0.0)	29 (0.8)	1 (0.4)	28 (0.9)
Congenital malformations, deformations and chromosomal abnormalities	11 (0.2)	1 (0.1)	10 (0.2)	2 (0.1)	0 (0.0)	2 (0.1)	9 (0.3)	1 (0.4)	8 (0.2)
Symptoms, signs and abnormal clinical and laboratory findings, not elsewhere Classified	1,766 (27.2)	211 (29.3)	1,555 (26.9)	827 (27.5)	120 (25.9)	707 (27.2)	939 (26.9)	91 (35.4)	848 (26.2)
Injury, poisoning and certain other consequences of external causes	749 (11.5)	95 (13.2)	654 (11.3)	373 (12.4)	70 (15.1)	303 (11.9)	376 (10.8)	25 (9.7)	351 (10.8)

#### Adverse Drug Events

Among the patients surveyed, 11.1% (720/6,504) visited the hospital and were hospitalized due to adverse drug events. The total number of adverse drug events that led to visits was 1,065, which included multiple adverse drug events at the time of hospital visits and hospitalization.

Table 3 shows the drug categories. The prevalence of adverse drug events was the highest for biological preparations (57.1%), followed by antineoplastic drugs (43.1%) and alkaloidal narcotics (21.6%). The prevalence of adverse drug events was 4.3% for over-the-counter drugs.

**TABLE 3:** Drug categories.

	All (n = 34,357)	Adverse drug events at the time of hospital visits and hospitalization		Suspected drugs (n = 1,227)	Prevalence of adverse drug events (%)
		Present	Absent		
		(n = 5,720)	(n = 28,637)		
Drugs used at the time of visit [n (%)]					
Agents affecting central nervous system	6,957 (20.2)	1,088 (19.0)	5,869 (20.5)	314	4.5
Agents affecting peripheral nervous system	261 (0.8)	40 (0.7)	221 (0.8)	8	0.7
Agents affecting sensory organs	163 (0.5)	14 (0.2)	149 (0.5)	0	0.0
Cardiovascular agents	6,514 (19.0)	1,188 (20.8)	5,326 (18.6)	203	3.1
Respiratory organ agents	2,229 (6.5)	192 (3.4)	2,037 (7.1)	9	0.4
Digestive organ agents	6,517 (19.0)	1,086 (19.0)	5,431 (19.0)	29	0.4
Hormones	896 (2.6)	199 (3.5)	697 (2.4)	53	5.9
Urogenital and anal organ agents	659 (1.9)	102 (1.8)	557 (1.9)	9	1.4
Other agents affecting individual organs	8 (0.0)	3 (0.1)	5 (0.0)	0	0.0
Vitamins	1,203 (3.5)	222 (3.9)	981 (3.4)	2	0.2
Nutrients, tonics	430 (1.3)	69 (1.2)	361 (1.3)	1	0.2
Blood and body fluid agents	1,739 (5.1)	378 (6.6)	1,361 (4.8)	190	15.5
Dialysis agents	1 (0.0)	0 (0.0)	1 (0.0)	0	0.0
Other agents affecting metabolism	2,304 (6.7)	385 (6.7)	1,919 (6.7)	87	3.8
Cellular function activating agents	3 (0.0)	1 (0.0)	2 (0.0)	0	0.0
Antineoplastic drugs	443 (1.3)	229 (4.0)	214 (0.7)	191	43.1
Allergic agents	1,322 (3.8)	115 (2.0)	1,207 (4.2)	8	0.6
Crude drugs	13 (0.0)	1 0.0	12 (0.0)	1	7.7
Traditional Chinese medicines	697 (2.0)	101 (1.8)	596 (2.1)	15	2.2
Other crude drugs and Chinese medicine formulations	9 (0.0)	1 (0.0)	8 (0.0)	0	0.0
Antibiotics	1,009 (2.9)	101 (1.8)	908 (3.2)	50	5.0
Chemotherapeutics	634 (1.8)	147 (2.6)	487 (1.7)	28	4.4
Biological preparations	14 (0.0)	9 (0.2)	5 (0.0)	8	57.1
Antiparasitic drugs	8 (0.0)	1 (0.0)	7 (0.0)	0	0.0
Dispensing medicines	18 (0.1)	2 (0.0)	16 (0.1)	0	0.0
Other agents not mainly for therapeutic purpose	8 (0.0)	3 (0.1)	5 (0.0)	1	12.5
Alkaloidal narcotics	37 (0.1)	16 (0.3)	21 (0.1)	8	21.6
Non-alkaloidal narcotics	26 (0.1)	7 (0.1)	19 (0.1)	0	0.0
Over-the-counter drugs	232 (0.7)	18 (0.3)	214 (0.7)	10	4.3

Table 4 shows the likely causality, classification, and severity of adverse drug events. Most events were “possible” (75.1%), classified as “gastrointestinal disorders” (25.4%), and “grade 2” (38.5%), respectively.

**TABLE 4:** Adverse drug events.

	All (n = 1,065)	Outpatients			Inpatients		
		All (n = 493)	Older adults (n = 247)	<65-year-olds (n = 246)	All (n = 572)	Older adults (n = 425)	<65-year-olds (n = 147)
	n (%)	n (%)	n (%)	n (%)	n (%)	n (%)	n (%)
A. Likelihood of causality							
Possible	800 (75.1)	432 (87.6)	216 (87.4)	216 (87.8)	368 (64.3)	271 (63.8)	97 (66.0)
Probable/likely	231 (21.7)	47 (9.5)	22 (8.9)	25 (10.2)	184 (32.2)	145 (34.1)	39 (26.5)
Certain	34 (3.2)	14 (2.8)	9 (3.6)	5 (2.0)	20 (3.5)	9 (2.1)	11 (7.5)
B. Classification							
Blood and lymphatic system disorders	31 (2.9)	4 (0.8)	2 (0.8)	2 (0.8)	27 (4.7)	21 (4.9)	6 (4.1)
Cardiac disorders	25 (2.3)	11 (2.2)	5 (2.0)	6 (2.4)	14 (2.4)	13 (3.1)	1 (0.7)
Congenital, familial and genetic disorders	0 (0.0)	0 (0.0)	0 (0.0)	0 (0.0)	0 (0.0)	0 (0.0)	0 (0.0)
Ear and labyrinth disorders	10 (0.9)	10 (2.0)	6 (2.4)	4 (1.6)	0 (0.0)	0 (0.0)	0 (0.0)
Endocrine disorders	0 (0.0)	0 (0.0)	0 (0.0)	0 (0.0)	0 (0.0)	0 (0.0)	0 (0.0)
Eye disorders	1 (0.1)	1 (0.2)	1 (0.4)	0 (0.0)	0 (0.0)	0 (0.0)	0 (0.0)
Gastrointestinal disorders	270 (25.4)	146 (29.6)	62 (25.1)	84 (34.1)	124 (21.7)	94 (22.1)	30 (20.4)
General disorders and administration site conditions	83 (7.8)	48 (9.7)	19 (7.7)	29 (11.8)	35 (6.1)	17 (4.0)	18 (12.2)
Hepatobiliary disorders	1 (0.1)	0 (0.0)	0 (0.0)	0 (0.0)	1 (0.2)	1 (0.2)	0 (0.0)
Immune system disorders	26 (2.4)	20 (4.1)	10 (4.0)	10 (4.1)	6 (1.0)	3 (0.7)	3 (2.0)
Infections and infestations	35 (3.3)	0 (0.0)	0 (0.0)	0 (0.0)	35 (6.1)	18 (4.2)	17 (11.6)
Injury, poisoning and procedural complications	72 (6.8)	34 (6.9)	27 (10.9)	7 (2.8)	38 (6.6)	36 8.5)	2 (1.4)
Investigations	145 (13.6)	28 (5.7)	13 (5.3)	15 (6.1)	117 (20.5)	80 (18.8)	37 (25.2)
Metabolism and nutrition disorders	103 (9.7)	37 (7.5)	25 (10.1)	12 (4.9)	66 (11.5)	54 (12.7)	12 (8.2)
Musculoskeletal and connective tissue disorders	3 (0.3)	2 (0.4)	0 (0.0)	2 (0.8)	1 (0.2)	1 (0.2)	0 (0.0)
Neoplasms benign, malignant and unspecified (incl cysts and polyps)	0 (0.0)	0 (0.0)	0 (0.0)	0 (0.0)	0 (0.0)	0 (0.0)	0 (0.0)
Nervous system disorders	130 (12.2)	92 (18.7)	41 (16.6)	51 (20.7)	38 (6.6)	32 (7.5)	6 (4.1)
Pregnancy, puerperium and perinatal conditions	0 (0.0)	0 (0.0)	0 (0.0)	0 (0.0)	0 (0.0)	0 (0.0)	0 (0.0)
Psychiatric disorders	14 (1.3)	5 (1.0)	2 (0.8)	3 (1.2)	9 (1.6)	5(1.2)	4 (2.7)
Renal and urinary disorders	26 (2.4)	0 (0.0)	0 (0.0)	0 (0.0)	26 (4.5)	24 (5.6)	2 (1.4)
Reproductive system and breast disorders	6 (0.6)	5 (1.0)	4 (1.6)	1 (0.4)	1 (0.2)	1 (0.2)	0 (0.0)
Respiratory, thoracic and mediastinal disorders	27 (2.5)	10 (2.0)	8 (3.2)	2 (0.8)	17 (3.0)	16 (3.8)	1 (0.7)
Skin and subcutaneous tissue disorders	35 (3.3)	24 (4.9)	9 (3.6)	15(6.1)	11 (1.9)	3 (0.7)	8 (5.4)
Social circumstances	0 (0.0)	0 (0.0)	0 (0.0)	0 (0.0)	0 (0.0)	0 (0.0)	0 (0.0)
Surgical and medical procedures	0 (0.0)	0 (0.0)	0 (0.0)	0 (0.0)	0 (0.0)	0 (0.0)	0 (0.0)
Vascular disorders	22 (2.1)	16 (3.2)	13 (5.3)	3 (1.2)	6 (1.0)	6 (1.4)	0 (0.0)
C. Severity							
Grade 1	241 (22.6)	170 (34.5)	71 (28.7)	99 (40.2)	71 (12.4)	49 (11.5)	22 (15.0)
Grade 2	410 (38.5)	272 (55.2)	147 (59.5)	125 (50.8)	138 (24.1)	99 (23.3)	39 (26.5)
Grade 3	301 (28.3)	40 (8.1)	23 (9.3)	17 (6.9)	261 (45.6)	190 (44.7)	71 (48.3)
Grade 4	110 (10.3)	11 (2.2)	6 (2.4)	5 (2.0)	99 (17.3)	84 (19.8)	15 (10.2)
Grade 5	3 (0.3)	0 (0.0)	0 (0.0)	0 (0.0)	3 (0.5)	3 (0.7)	0 (0.0)

#### Direct Costs for Management of Adverse Drug Events

Table 5 shows the direct costs of managing adverse drug events in outpatients. The direct costs per patient who visited the hospital due to adverse drug events (*C*_*out, ade*_) were 21,281 and 22,590 yen (166 and 176 euros as on September 13, 2021) for all ages and older adults, respectively.

**TABLE 5:** Direct costs for management of adverse drug events (all ages, older adults and < 65 year-olds).

	All (n = 361)	Older adults (n = 191)	<65 year-olds (n = 170)
A. Outpatients			
The direct costs per patient who visited the hospital due to adverse drug events [*C*_*out, ade*_] (yen)	21,281 (≅166 euros)	22,590 (≅176 euros)	19,809 (≅154 euros)
The direct cost per patient who visited the hospital regardless of the visit reasons [*C*_*out, targ*_] (yen)	1,919 (≅15 euros)	2,925 (≅23 euros)	1,332 (≅10 euros)
The direct costs in the whole country [*C*_*out, nat*_] (yen/year)	115.73 billion (≅902 million euros)	59.69 billion (≅465 million euros)	55.47 billion (≅432 million euros)

	All (n = 359)	Older adults (n = 272)	<65 year-olds (n = 87)
B. Inpatients			
The direct costs per patient who admitted to the hospital due to adverse drug events [*C*_*in, ade*_] (yen)	853,175 (≅6,648 euros)	874,582 (≅6,815 euros)	786,247 (≅9,127 euros)
The direct costs per patient who was admitted to the hospital regardless of the reasons [*C*_*in, targ*_] (yen)	122,467 (≅954 euros)	154,874 (≅1,207 euros)	70,884 (≅552 euros)
The direct costs in the whole country [*C*_*in, nat*_] (yen/year)	688.80 billion (≅5,367 million euros)	537.50 billion (≅4,188 million euros)	140.40 billion (≅1,094million euros)

For all ages, the direct cost per patient who visited the hospital regardless of the visit reasons (*C*_*out, targ*_) was 1,919 yen (15 euros), and the direct cost in the whole country (*C*_*out, nat*_) was 115.73 billion yen (902 million euros) per year. For older adults, the direct cost per patient who visited the hospital regardless of the visit reasons (*C*_*out, targ*_) was 2,925 yen (23 euros), and the direct cost in the whole country (*C*_*out, nat*_) was 59.69 billion yen (465 million euros) per year.

Table 5 shows the direct costs of managing adverse drug events in inpatients. The direct costs per patient admitted to the hospital due to adverse drug events (*C*_*in, ade*_) were 853,175 and 874,582 yen (6,648 and 6,815 euros) for all ages and older adults, respectively.

For all ages, the direct cost per patient admitted to the hospital regardless of hospitalization reasons (*C*_*in, targ*_) was 122,467 yen (954 euros) and that of the whole country (*C*_*in, nat*_) was 688.80 billion yen (5,367 million euros) per year. For older adults, the direct cost per patient who was admitted to the hospital regardless of the hospitalization reasons (*C*_*in, targ*_) was 154,874 yen (1,207 euros), and at the country level, it was (*C*_*in, nat*_) was 537.50 billion yen (4,188 million euros) per year.

In sum, combining inpatients and outpatients, the direct costs of managing adverse drug events were 804.53 billion yen (6,269 million euros) per year for all ages and 597.19 billion yen (4,654 million euros) per year for older ages for the whole country.

### Analysis of Patients Who Used the Drugs Listed in the BCJ and GMTSE-2015

#### Patient Characteristics

Table 6 shows the background of older adults who used the drugs listed in the BCJ and GMTSE-2015. The most common diseases among outpatients according to the BCJ were related to the circulatory system (90.2%), and those according to GMTSE-2015 were related to the digestive system (88.0%). The prevalence of adverse drug events in outpatients was 3.3% (17/519) and 5.6% (59/1,045) according to the BCJ and GMTSE-2015, respectively. The most common diseases among inpatients, according to both the BCJ and the GMTSE-2015, were related to the circulatory system (77.8 and 76.5%, respectively). The prevalence of adverse drug events in inpatients was 4.8% (23/481) and 9.2% (107/1,159) according to the BCJ and GMTSE-2015, respectively.

**TABLE 6:** Background of patients with the drugs listed in the Beers Criteria Japanese version and “Guidelines for medical treatment and its safety in the elderly 2015”.

		BCJ			GMTSE-2015	
	All (n = 519)	Adverse drug events at the time of hospital visits		All (n = 1,045)	Adverse drug events at the time of hospital visits	
		Present (n = 17)	Absent (n = 502)		Present (n = 59)	Absent (n = 986)
A. Outpatients					
Sex [n (%)]					
Male	224 (43.2)	8 (47.1)	216 (43.0)	476 (45.6)	28 (47.5)	448 (45.4)
Female	295 (56.8)	9 (52.9)	286 (57.0)	569 (54.4)	31 (52.5)	538 (54.6)
Age (average ± standard deviation)	77.7 ± 6.8	79.0 ± 6.6	77.7 ± 6.8	78.2 ± 7.2	79.3 ± 7.7	78.2 ± 7.1
Number of drugs used (average±standard deviation)	8.2 ± 4.1	7.8 ± 2.8	8.3 ± 4.1	7.8 ± 4.2	8.2 ± 4.2	7.8 ± 4.2
Disease [n (%)]					
Certain infectious and parasitic diseases	166 (32.0)	2 (11.8)	164 (32.7)	337 (32.2)	14 (23.7)	323 (32.8)
Neoplasms	125 (24.1)	3 (17.6)	122 (24.3)	308 (29.5)	10 (16.9)	298 (30.2)
Diseases of the blood and blood-forming organs and certain disorders involving the immune mechanism	173 (33.3)	2 (11.8)	171 (34.1)	361 (34.5)	15 (25.4)	346 (35.1)
Endocrine, nutritional and metabolic diseases	393 (75.7)	13 (76.5)	380 (75.7)	799 (76.5)	46 (78.0)	753 (76.4)
Mental and behavioral disorders	256 (49.3)	9 (52.9)	247 (49.2)	421 (40.3)	26 (44.1)	395 (40.1)
Diseases of the nervous system	319 (61.5)	8 (47.1)	311 (62.0)	578 (55.3)	30 (50.8)	548 (55.6)
Diseases of the eye and adnexa	266 (51.3)	9 (52.9)	257 (51.2)	491 (47.0)	24 (40.7)	467 (47.4)
Diseases of the ear and mastoid process	94 (18.1)	3 (17.6)	91 (18.1)	186 (17.8)	9 (15.3)	177 (18.0)
Diseases of the circulatory system	468 (90.2)	13 (76.5)	455 (90.6)	908 (86.9)	49 (83.1)	859 (87.1)
Diseases of the respiratory system	320 (61.7)	9 (52.9)	311 (62.0)	654 (62.6)	36 (61.0)	618 (62.7)
Diseases of the digestive system	459 (88.4)	15 (88.2)	444 (88.4)	920 (88.0)	51 (86.4)	869 (88.1)
Diseases of the skin and subcutaneous tissue	252 (48.6)	6 (35.3)	246 (49.0)	504 (48.2)	29 (49.2)	475 (48.2)
Diseases of the musculoskeletal system and connective tissue	314 (60.5)	7 (41.2)	307 (61.2)	602 (57.6)	29 (49.2)	573 (58.1)
Diseases of the genitourinary system	317 (61.1)	12 (70.6)	305 (60.8)	600 (57.4)	30 (50.8)	570 (57.8)
Pregnancy, childbirth and the puerperium	0 (0.0)	0 (0.0)	0 (0.0)	0 (0.0)	0 (0.0)	0 (0.0)
Certain conditions originating in the perinatal period	0 (0.0)	0 (0.0)	0 (0.0)	0 (0.0)	0 (0.0)	0 (0.0)
Congenital malformations, deformations and chromosomal abnormalities	2 (0.4)	0 (0.0)	2 (0.4)	2 (0.2)	0 (0.0)	2 (0.2)
Symptoms, signs and abnormal clinical and laboratory findings, not elsewhere classified	297 (57.2)	9 (52.9)	288 (57.4)	590 (56.5)	33 (55.9)	557 (56.5)
Injury, poisoning and certain other consequences of external causes	73 (14.1)	3 (17.6)	70 (13.9)	150 (14.4)	13 (22.0)	13 (13.9)

		BCJ			GMTSE-2015	
	All (n = 481)	Adverse drug events at the time of hospital visits		All (n = 1,159)	Adverse drug events at the time of hospital visits	
		Present (n = 23)	Absent (n = 458)		Present (n = 107)	Absent (n = 1,052)
B. Inpatients					
Sex [n (%)]					
Male	238 (49.5)	12 (52.2)	226 (49.3)	624 (53.8)	58 (54.2)	566 (53.8)
Female	243 (50.5)	11 (47.8)	232 (50.7)	535 (46.2)	49 (45.8)	486 (46.2)
Age (average ± standard deviation)	78.4 ± 7.6	81.4 ± 8.5	78.2 ± 7.5	78.7 ± 7.8	80.3 ± 7.6	78.5 ± 7.8
Number of drugs used (average ± standard deviation)	9.3 ± 4.3	9.3 ± 3.7	9.3 ± 4.3	8.3 ± 4.0	9.7 ± 4.2	8.1 ± 3.9
Length of hospitalization [days, median (range)]	15 (1–187)	15 (4–65)	15 (1–187)	15 (1–242)	14 (1–65)	16(1–242)
Disease [n (%)]					
Certain infectious and parasitic diseases	64 (13.3)	1 (4.3)	63 (13.8)	160 (13.8)	4 (3.7)	156 (14.8)
Neoplasms	106 (22.0)	2 (8.7)	104 (22.7)	270 (23.3)	19 (17.8)	251 23.9)
Diseases of the blood and blood-forming organs and certain disorders involving the immune mechanism	60 (12.5)	5 (21.7)	55 (12.0)	159 (13.7)	21 (19.6)	138 (13.1)
Endocrine, nutritional and metabolic diseases	211 (43.9)	11 (47.8)	200 (43.7)	564 (48.7)	66 (61.7)	498 (47.3)
Mental and behavioural disorders	51 (10.6)	3 (13.0)	48 (10.5)	84(7.2)	7 (6.5)	77 (7.3)
Diseases of the nervous system	61 (12.7)	1 (4.3)	60 (13.1)	148 (12.8)	15 (14.0)	133 (12.6)
Diseases of the eye and adnexa	75 (15.6)	5 (21.7)	70 (15.3)	153 (13.2)	15 (14.0)	138 (13.1)
Diseases of the ear and mastoid process	9 (1.9)	0 (0.0)	9 (2.0)	17 (1.5)	1 (0.9)	16 (1.5)
Diseases of the circulatory system	374 (77.8)	19 (82.6)	355 (77.5)	887 (76.5)	98 (91.6)	789 (75.0)
Diseases of the respiratory system	142 (29.5)	3 (13.0)	139 (30.3)	337 (29.1)	20 (18.7)	317 (30.1)
Diseases of the digestive system	251 (52.2)	10 (43.5)	241 (52.6)	592 (51.1)	68 (63.6)	524 (49.8)
Diseases of the skin and subcutaneous tissue	41 (8.5)	1 (4.3)	40 (8.7)	93 (8.0)	4 (3.7)	89 (8.5)
Diseases of the musculoskeletal system and connective tissue	91 (18.9)	5 (21.7)	86 (18.8)	224 (19.3)	20 (18.7)	204 (19.4)
Diseases of the genitourinary system	137 (28.5)	6 (26.1)	131 (28.6)	342 (29.5)	40 (37.4)	302 (28.7)
Pregnancy, childbirth and the puerperium	0 (0.0)	0 (0.0)	0 (0.0)	0 (0.0)	0 (0.0)	0(0.0)
Certain conditions originating in the perinatal period	0 (0.0)	0 (0.0)	0 (0.0)	0 (0.0)	0 (0.0)	0 (0.0)
Congenital malformations, deformations and chromosomal abnormalities	0 (0.0)	0 (0.0)	0 (0.0)	0 (0.0)	0 (0.0)	0 (0.0)
Symptoms, signs and abnormal clinical and laboratory findings, not elsewhere classified	18 (4.3)	1 (4.3)	37 (3.7)	37 (3.2)	5 (4.7)	32 (3.0)
Injury, poisoning and certain other consequences of external causes	60 (52.2)	12 (52.2)	138 (10.5)	138 (11.9)	23 (21.5)	115 (10.9)

BCJ, the Beers Criteria Japanese version; GMTSE-2015, “Guidelines for medical treatment and its safety in the elderly 2015”.

#### Calculation of Cost of Avoidable Adverse Drug Events in Older Adults

Table 7 shows the direct cost of avoidable adverse drug events in older adults based on the BCJ and the GMTSE-2015. For the patients using drugs listed in the BCJ and GMTSE-2015, the direct costs of avoidable adverse drug events per patient (*C*_*out, targ, BCJ*_ and *C*_*out, targ, GMTSE*_) were 3,212 and 3,341 yen (25 and 26 euros) for outpatients and the direct costs (*C*_*in, targ, BCJ*_ and *C*_*in, targ, GMTSE*_) were 55,548 and 80,246 yen (433 and 625 euros) for inpatients, respectively. Meanwhile, the direct costs for the whole country per year (*C*_*out, nat, BCJ*_ and *C*_*out, nat, GMTSE*_) were 23.06 billion and 48.30 billion yen (180 million and 376 million euros) for outpatients and the direct costs (*C*_*in, nat, BCJ*_ and *C*_*in, nat, GMTSE*_) were 60.37 billion and 210.14 billion yen (470 and 1,637 million euros) for inpatients, respectively.

**TABLE 7:** Direct costs of avoidable adverse drug events in older adults.

A. Outpatients		
	**Patients using drugs listed in BCJ**	**Patients using drugs listed in GMTSE-2015**
The direct costs per patient who was older adults using the drugs listed regardless of the visit reasons (yen)	3,212 (≅25 euros)[*C*_*out, targ, BCJ*_]	3,341 (≅26 euros) [*C*_*out, targ, GMTSE*_]
The direct cost in the whole country (yen/year)	23.06 billion (≅180 million euros) [*C*_*out, nat, BCJ*_]	48.30 billion (≅376 million euros) [*C*_*out, nat, GMTSE*_]
**B. Inpatients**		
	**Patients using drugs listed in BCJ**	**Patients using drugs listed in GMTSE-2015**
The direct costs per patient who was older adults using the drugs listed regardless of the hospitalization reasons (yen)	55,548 (≅433 euros) [*C*_*in, targ, BCJ*_]	80,246 (≅625 euros) [*C*_*out, targ, GMTSE*_]
The direct cost in the whole country (yen/year)	60.37 billion (≅470 million euros) [*C*_*in, nat, BCJ*_]	210.14 billion (≅1,637 million euros) [*C*_*in, nat, GMTSE*_]

BCJ, the Beers Criteria Japanese version; GMTSE-2015, “Guidelines for medical treatment and its safety in the elderly 2015”.

On combining inpatients and outpatients, the direct cost of avoidable adverse drug events in older adults was 83.43–258.44 billion yen (650–2,014 million euros) per year for the whole country.

## Discussion

We conducted a retrospective survey to evaluate the direct cost of managing adverse drug events and the potential reduction of direct costs using the BCJ and GMTSE-2015.

Adverse drug events were the reason for 9.0% of all outpatient visits in Japan. This is consistent with the findings of various other studies reporting that adverse drug events are the reason for 5.5–53% of outpatient visits (Hanlon et al., 1997; Honigman et al., 2001; Gandhi et al., 2003). Furthermore, they accounted for 14.4% of all hospitalizations in this study, which is higher than the finding of another Japanese study that reported only 1.7% of hospitalizations owing to adverse drug events (Koinuma et al., 2006). This is probably because the report by Koinuma was limited to adverse drug events due to inappropriate prescription. Further, the number of visits due to adverse drug events caused by antineoplastic drugs increased with outpatient cancer chemotherapy. However, the current findings are in line with various reports worldwide; that is, the rate of hospitalization due to adverse drug events ranges from 1.3 to 30.4% (Dartnell et al., 1996; Chan et al., 2001; Onder et al., 2002; Wawruch et al., 2009; Ruiter et al., 2012; Parameswaran Nair et al., 2017).

During a medical economic assessment, direct costs of medical resources are estimated using the so-called micro-costing method (Gold et al., 1996; Drummond et al., 2005). The micro-costing method calculates the sum of the direct costs of each clinic/technical fee including medical supplies and drugs. Japan’s public medical insurance system is mainly based on the conception of the micro-costing method. In Japan’s public medical insurance system, outpatient costs are paid through fees-for-service whereas inpatient costs are paid for by the coexistence of package pricing and fee-for-service. In this study, we evaluated the direct costs from the perspective of medical cost payers under the public medical insurance system. From this perspective, the direct cost of managing adverse drug events corresponds to the total costs payable by patients and/or health insurance association pertaining to the hospital visit and hospitalization caused by adverse drug events. The direct cost of managing adverse drug events considered to be 0 yen when the reason for the hospital visit and hospitalization is not due to adverse drug events.

The direct cost per patient who visited the hospital due to adverse drug events was 21,281 yen, which is somewhat similar to that of Germany (381 euros, that is, 48,893 yen) (Stark et al., 2011). In contrast, the direct cost per patient admitted to the hospital due to adverse drug events was 853,175 yen. Meanwhile, in the Netherlands, Spain, and Germany, the direct cost per patient admitted to the hospital due to adverse drug events was 6,009 euros, 3,857–4,656 euros, and 2,250 euros (771,134 yen, 494,968–597,504 yen, 288,743 yen, respectively) (Carrasco-Garrido et al., 2010; Leendertse et al., 2011; Rottenkolber et al., 2011). Although this study’s result is slightly similar to that of these previous studies, it is higher than that of the German report, probably because the latter was limited to internal medicine inpatients. In addition, the methods for calculating hospitalization cost in Japan differ from those in other countries.

In this study, which combined inpatients and outpatients, the direct costs of managing adverse drug events were 804.53 billion yen (6,269 million euros) per year for all ages in Japan. In the Netherlands, the direct cost of managing adverse drug events in inpatients was about 94 million euros (12 billion yen) annually in 2011(Leendertse et al., 2011). In Spain, this annual cost was about 226–273 million euros (29–35 billion yen) for inpatients in 2010 (Carrasco-Garrido et al., 2010), while in Germany it was 816 million euros (105 billion yen) for outpatients in 2011 (Stark et al., 2011). Because the medical and technical fee differs in each country, the calculated costs cannot be accurately compared each other. However, the cost in Japan in this study was slightly high compared with those countries when the costs were corrected according to total population of those countries. That would be due to a larger population of older people in Japan than in those countries.

Older people are more likely to experience adverse drug events due to decreased physiological function (Mangoni and Jackson, 2004). Risk factors for adverse drug events in older adults include polypharmacy, dementia, reduced visual acuity, renal damage, and liver damage (The Japan Geriatrics Society, 2015). In particular, polypharmacy is an important risk factor of adverse drug events resulting in outpatient visits and hospitalization (Matsuyama et al., 2021). To prevent adverse drug events, the BCJ and GMTSE-2015 have begun to be used to suggest appropriate prescription drugs, reduce the use of drugs with a high risk of adverse events, and propose alternative drugs with lower risk. In this study, we calculated the direct cost of avoidable adverse drug events using the BCJ and GMTSE-2015 as references.

For the patients using drugs listed in the BCJ and GMTSE-2015, the direct costs of managing adverse drug events per patient were 3,212–3,341 and 55,548–80,246 yen (25–26 and 433–625 euros) for outpatients and inpatients, respectively. Meanwhile, the direct costs for the entire country per year were 23.06–48.30 and 60.37–210.14 billion yen (180–376 and 470–1,637 million euros) for outpatients and inpatients, respectively. A previous study of patients who used the drugs listed in the BCJ and GMTSE-2015 showed that the direct cost of managing adverse drug events per patient was 497–798 and 1,109–13,371 yen (4–6 and 9–104 euros) for outpatients and inpatients, respectively, and the direct costs for the whole country per year were 267.87–381.42 and 2.18–79.42 billion yen (2,087–2,972 and 17–619 million euros) for outpatients and inpatients, respectively (Tachi et al., 2019).

Medical costs for older adults are 25,127.6 billion yen (196 billion euros) per year in Japan (Ministry of Health, Labour and Welfare in Japan, 2015), while the direct costs of managing adverse drug events in older adults were 804.53 billion yen (6,269 million euros) for all ages, suggesting that the direct costs account for a certain proportion of the medical cost in Japan.

The proportion of the direct costs in the whole country for patients with adverse drug events due to drugs listed in the BCJ and GMTSE-2015 to those due to all kinds of drugs was 14.0% (83.43/597.19) to 43.3% (258.44/597.19) in older adults (outpatients and inpatients). A previous study in Japan indicates that polypharmacy is an induction risk factor of adverse drug events resulting in outpatient visits and hospitalization (Matsuyama et al., 2021). Therefore, adverse drug events resulting in outpatient visits and hospitalization should be avoided through prescription optimization and changing, reducing, and discontinuing the BCJ- and GMTSE-2015-listed drugs as needed. Of course, not all calculated costs of the adverse events through the BCJ and GMTSE-2015 would be reduced in actuality because other costs such as the lack of therapy and other adverse events caused by substitute drugs might occur due to the changed, reduced or discontinued prescription. Cost reduction based on the prevention of adverse drug events in older adults through the BCJ and GMTSE-2015 would be one of practical approach to reduce the costs of adverse drug events and eventually, reduce overall medical costs.

This study’s limitation is that it is a retrospective survey using electronic medical records that was conducted at only one general hospital in one region. The number of patients included in this study was small compared to that in the study using dataset of health insurance and claims. However, the smaller sample included detailed background of patients and adverse drug events, which are not available in the dataset of health insurance and claims. In addition, in this study, the extrapolation of the cost in the sample to the national cost was rough. To resolve the ensuring problems, Slight et al. reported a better and more defendable approach, which involves estimating the population-adjusted costs per patient and population-adjusted adverse drug events nationally, followed by an extrapolation (Slight et al., 2018). In Japan, the public medical insurance system covers the medical care of the whole nation and the Japanese government does not publicize the dataset of attributes of outpatients and inpatients of the whole nation. Therefore, we could not adjust extrapolation, for example, through multi-variate analysis. However, we consider the unadjusted extrapolation acceptable in this study for the three reasons below. First, from the patient survey in 2014 (Ministry of Health, Labour and Welfare in Japan, 2014), there are few differences of attributes in the data in this study and the whole nation. Second, the whole nation in Japan can receive the same quality of medical care with the same fee. Third, the hospital in this study is a representative hospital in Japan because it has almost all medical departments and gives both primary and secondary medical cares.

## Conclusion

We found that, in Japan, high medical costs are often due to managing adverse drug events, and that the costs of avoidable adverse drug events—based on the BCJ and GMTSE-2015—in older adults account for a substantial proportion of overall medical costs. By using BCJ and GMTSE-2015 as references for medicines that should not be prescribed to older adults, it may be possible to avoid adverse drug events and reduce medical costs may be possible.

## Data Availability

The raw data supporting the conclusions of this article will be made available by the authors, without undue reservation.
