# Supplementary material for: Evaluation of the Direct Costs of Managing Adverse Drug Events in all Ages and of Avoidable Adverse Drug Events in Older Adults in Japan
Source: Front Pharmacol. 2021 Nov 17;12:761607. doi: 10.3389/fphar.2021.761607 (PMC8635725; doi:10.3389/fphar.2021.761607)
Supplement: Supplementary file 1 [file DataSheet1.docx]

Supplementary Material

# Supplementary Data

***1. Direct costs of managing adverse drug events in outpatients***

We obtained the **direct** costs of managing adverse drug events in outpatients in the whole country (*C_out, nat_*) using Equation 1. The **direct** costs were extrapolated to the national level (whole country) based on the overview of the 2014 patient survey (Ministry of Health, Labour and Welfare in Japan, 2014). For outpatients, the total number of outpatients (7,238.4 thousand, *N_out, nat_*) minus the total number of 0-year-old outpatients (68.3 thousand, *N_out, nat, 0y_*) taken from the “estimated number of patients by type of facility by age group” data category in the 2014 patient survey overview (Ministry of Health, Labour and Welfare in Japan, 2014) was 7,170.1 thousand (*N_out, nat_ − N_out, nat, 0y_*). We then multiplied this number (7,170.1 thousand) by 365 days to obtain the number of outpatients aged 1 year or older who visited clinics/hospitals nationwide in one year [2,617,086,500, (*N_out, nat_ − N_out, nat, 0y_*) × 365]. Based on the number of outpatients with more than one drug used during their hospital visit without reservation (4,003, *N_out, targ_*) against the number of outpatients aged 1 year or older who visited Gifu Municipal Hospital between July 1 and December 31, 2015 (173,710, *N_out, hosp_*), we estimated the *nationwide* number of outpatients with more than one drug used at hospital visits without reservation [60,308,544, (*N_out, nat_ − N_out, nat, 0y_*) × 365 × *N_out, targ_* / *N_out, hosp_*]. The **direct** cost in the whole country (*C_out, nat_*) was calculated by multiplying the number of outpatients with more than one drug used during their hospital visit without reservation nationwide (60,308,544) by the **direct** cost of managing adverse drug events per patient (*C_out, targ_*).

***2. Direct costs of managing adverse drug events in inpatients***

We obtained the **direct** costs of managing adverse drug events in inpatients in the whole country (*C_in, nat_*) using Equation 2. The **direct** costs were extrapolated to the national level based on the overview of the 2014 patient survey (Ministry of Health, Labour and Welfare in Japan, 2014). The total number of inpatients (1,318.8 thousand, *N_in, nat_*) minus “the total number of 0-year-olds (10.8 thousand, *N_in, nat, 0y_*) taken from the “estimated number of patients by type of facility by age group” data category in the 2014 patient survey overview (Ministry of Health, Labour and Welfare in Japan, 2014) was 1308.0 thousand (*N_in, nat_ − N_in, nat, 0y_*). We then divided this number (1308.0 thousand) by the total number (31.9 days) of days in an “average hospital stay of discharge patients by age group according to injury/illness classification” (Ministry of Health, Labour and Welfare in Japan, 2014) and multiplied it by 365 days to obtain the nationwide number of inpatients aged 1 year or older in one year [14,966,144, (*N_in, nat_ − N_in, nat, 0y_*) × 365/31.9]. Based on the number of inpatients with more than one drug used during hospitalization without reservation (2,501, *N_in, targ_*) against the number of inpatients aged 1 year or older who were admitted to Gifu Municipal Hospital between July 1 and December 31, 2015 (6,655, *N_in, hosp_*), we estimated the nationwide number of inpatients with more than one drug used during hospitalization without reservation [5,624,392, (*N_in, nat_ − N_in, nat, 0y_*) × 365/31.9 × *N_in, targ_* / *N_in, hosp_*]. The direct cost in the whole country (*C_in, nat_*) was calculated by multiplying the nationwide number of inpatients with more than one drug used at hospitalization without reservation (5,624,392) by the **direct** cost for **management** of adverse drug events per patient (*C_in, targ_*).

***3. Direct cost of avoidable adverse drug events in older outpatients***

We obtained the **direct** costs of avoidable adverse drug events **based on the BCJ and GMTSE-2015** in older outpatients in the whole country (*C_out, nat, BCJ_* and *C_out, nat, GMTSE_*) using Equations 3 and 4. The **direct** costs were extrapolated to the national level based on the 2014 patient survey overview (Ministry of Health, Labour and Welfare in Japan, 2014). We then estimated the number of outpatients aged 65 years or older who visited clinics/hospitals nationwide in one year (1,281,223,000, *N_out, nat, older_* × 365) by multiplying the number of over-65-year-old outpatients (3,510.2 thousand, *N_out, nat, older_*) (see the “estimated number of patients by type of facility by age group” data category in the overview of the 2014 patient survey (Ministry of Health, Labour and Welfare in Japan, 2014) by 365 days. Based on the number of older outpatients using more than one drugs listed in the **BCJ and GMTSE-2015** during a hospital visit without reservation (519 and 1,045, *N_out, targ, BCJ_* and *N_out, targ, GMTSE_*, respectively) against the number of older outpatients who visited Gifu Municipal Hospital between July 1 and December 31, 2015 (92,611, *N_out, hosp, older_*), we estimated the nationwide number of outpatients using more than one drug listed in the **BCJ and GMTSE-2015** during hospital visits without reservation (7,180,084, *N_out, nat, older_* × 365 × *N_out, targ, BCJ_* / *N_out, hosp, older_* and 14,457,009, *N_out, nat, older_* × 365 × *N_out, targ, GMTSE_* / *N_out, hosp, older_,* respectively). The **direct** cost in the whole country (*C_out, nat, BCJ_* and *C_out, nat, GMTSE_*) was calculated by multiplying the nationwide number of outpatients using more than one drug listed in the **BCJ and GMTSE-2015** during hospital visits without reservation (7,180,084 and 14,457,009, respectively) by the direct cost of avoidable adverse drug events per patient who used the drugs listed in the **BCJ and GMTSE-2015** (*C_out, targ, BCJ_* and *C_out, targ, GMTSE_*).

***4. Direct cost of avoidable adverse drug events in older inpatients***

We obtained the **direct** costs of **avoidable** adverse drug events **based on the BCJ and GMTSE-2015** in older inpatients in the whole country (*C_in, nat, BCJ_* and *C_in, nat, GMTSE_*) using Equations 5 and 6. The **direct** costs were extrapolated to the national level based on the 2014 patient survey overview (Ministry of Health, Labour and Welfare in Japan, 2014). We then estimated the nationwide number of inpatients aged 65 year or older in one year (8,204,185, *N_in, nat, older_* × 365/41.7) by dividing the number of over-65-year-old inpatients (937.3 thousand, *N_in, nat, older_*) (see the “estimated number of patients by type of facility by age group” data category in the 2014 patient survey (Ministry of Health, Labour and Welfare in Japan, 2014) by the total number (41.7 days) of days in an “average hospital stay of discharge patients by age group according to injury/illness classification” (Ministry of Health, Labour and Welfare in Japan, 2014) and then multiplying it by 365 days. Based on the number of older inpatients using more than one drug listed in the **BCJ and GMTSE-2015** at hospitalization without reservation (481 and 1,159, *N_in, targ, BCJ_* and *N_in, targ, GMTSE_*, respectively) against the number of older patients who were admitted to Gifu Municipal Hospital between July 1 and December 31, 2015 (3,631, *N_in, hosp, older_*), we estimated the number of inpatients using more than one drug listed in the **BCJ and GMTSE-2015** at hospitalization without reservation nationwide (1,085,616, *N_in, nat, older_* × 365/41.7 × *N_in, targ, BCJ_* / *N_in, hosp, older_* and 2,615,860, *N_in, nat, older_* × 365/41.7 × *N_in, targ, GMTSE_* / *N_in, hosp, older_*, respectively). The **direct** cost in the whole country (*C_in, nat, BCJ_* and *C_in, nat, GMTSE_*) was calculated by multiplying the number of inpatients using more than one drug listed in the **BCJ and GMTSE-2015** at hospitalization without reservation nationwide (7,180,084 and 14,457,009, respectively) by the direct cost of avoidable adverse drug events per patient who used the drugs listed in the **BCJ and GMTSE-2015** (*C_in, targ, BCJ_* and *C_in, targ, GMTSE_*).
